# Photoplethysmography Signal Wavelet Enhancement and Novel Features Selection for Non-Invasive Cuff-Less Blood Pressure Monitoring

**DOI:** 10.3390/s23042321

**Published:** 2023-02-19

**Authors:** Filippo Attivissimo, Luisa De Palma, Attilio Di Nisio, Marco Scarpetta, Anna Maria Lucia Lanzolla

**Affiliations:** Department of Electrical and Information Engineering, Polytechnic University of Bari, 70125 Bari, Italy

**Keywords:** blood pressure (BP), hypertension, features selection, maximal overlap discrete wavelet transform (MODWT), photoplethysmography (PPG), telemedicine

## Abstract

In this paper, new features relevant to blood pressure (BP) estimation using photoplethysmography (PPG) are presented. A total of 195 features, including the proposed ones and those already known in the literature, have been calculated on a set composed of 50,000 pulses from 1080 different patients. Three feature selection methods, namely Correlation-based Feature Selection (CFS), RReliefF and Minimum Redundancy Maximum Relevance (MRMR), have then been applied to identify the most significant features for BP estimation. Some of these features have been extracted through a novel PPG signal enhancement method based on the use of the Maximal Overlap Discrete Wavelet Transform (MODWT). As a matter of fact, the enhanced signal leads to a reliable identification of the characteristic points of the PPG signal (e.g., systolic, diastolic and dicrotic notch points) by simple means, obtaining results comparable with those from purposely defined algorithms. For systolic points, mean and std of errors computed as the difference between the locations obtained using a purposely defined already known algorithm and those using the MODWT enhancement are, respectively, 0.0097 s and 0.0202 s; for diastolic points they are, respectively, 0.0441 s and 0.0486 s; for dicrotic notch points they are 0.0458 s and 0.0896 s. Hence, this study leads to the selection of several new features from the MODWT enhanced signal on every single pulse extracted from PPG signals, in addition to features already known in the literature. These features can be employed to train machine learning (ML) models useful for estimating systolic blood pressure (*SBP*) and diastolic blood pressure (*DBP*) in a non-invasive way, which is suitable for telemedicine health-care monitoring.

## 1. Introduction

Nowadays, the importance of and need for adequate monitoring of vital signs with telemedicine solutions at home and for pre-hospital and intrahospital care is increasing, with the aim of ensuring the early identification and prevention of cardiovascular and other diseases. In recent years, telemedicine has become increasingly pervasive, thanks to the use of innovative wearable sensors, miniaturized devices and even smartphones that allow the monitoring of vital signs and which are simple to use, non-invasive, and wireless [1,2,3,4,5,6].

Among vital signs, the monitoring of blood pressure (BP) is a very important aspect in the treatment of many clinical conditions; it is relevant for the assessment of the state of hypertension, which is associated with chronic diseases and an increase in mortality and morbidity. Currently, measurements are made using cuff-based devices that are complicated, not always accurate and are prone to errors if the cuff is not of the correct size or if the appropriate calibrations are not made; hence, a fundamental prerequisite is that both the caregiver and the patient need to be trained in their use. Moreover, the gold standard is the invasive blood pressure monitoring of arterial blood pressure (ABP), which is carried out through the cannulation of a peripheral artery and is utilized in the management of critically ill and perioperative patients.

A measurement technique that can be exploited and is increasingly adopted for real-time monitoring of vital signs is plethysmography (PG). There are different types of plethysmography, such as those based on capacitive or inductive properties, piezoelectric properties [7], or those exploiting optical properties such as photoplethysmography (PPG). PPG measures light transmitted or reflected between a source (LED) and a photodetector placed on the surface of the skin, which is affected by volumetric changes in blood circulation [8]. In transmission measurements, it is possible to use red light (680 nm) or near infrared light (810 nm) as they allow a deeper penetration. In reflection measurements, the photodetector reveals backscattered or reflected light from tissues, bones, and blood vessels [9]. Since the maximum pulsatile component of reflected light occurs in the wavelength range between 510 nm and 590 nm, green light (565 nm) and yellow light (590 nm) can be used [10,11].

The PPG signal is characterized by a pulsatile part and a steady part; the pulsatile one is associated with the variation of blood pressure with blood volume changes, and it is synchronous to the pulse, while the steady one is associated with some aspects such as respiration and the sympathetic nervous system.

PPG is usually used to measure oxygen saturation (SpO_2_), to estimate HR and respiration rate and to evaluate atherosclerosis and arterial stiffness. Moreover, the extension of PPG to the estimation of BP, with appropriate calibration, is also an active research area. Indeed, the ability to use, for these purposes, a signal acquired in a single site by a wearable, non-invasive, wireless, miniaturized, inexpensive and easy to use sensor certainly has a great potential to control the health of an individual and for detecting states of hypertension; in fact, nowadays, the interest in the use of the PPG signal is becoming more and more important for the estimation of BP.

Many studies have focused over the years and even today on the use of electrocardiogram (ECG) and PPG signals acquired simultaneously for the estimation of BP [12], but also in the use of only the PPG signal; in fact, there is a relationship between the PPG signal and BP that is related to a measure of the speed of the blood flow, known as pulse wave velocity (PWV) [13], so it is possible to measure the time for a blood pulse to reach a peripheral point from the heart; that is, the pulse transit time (PTT) [14]. A shorter PTT is associated with a higher BP, and a longer PTT with a lower BP. However, this approach requires multi-site acquisition and the need for synchronization between the two signals, so the use of the PPG signal alone is certainly an approach with a great potential to be pursued and improved; in fact, it can simplify the acquisition, but requires more complex processing for obtaining BP from the PPG signal. Moreover, PPG for the estimation of BP is not free from criticalities and limitations, such as noise elimination, development of multi-photodetectors, event detection, the need to carry out individual calibration based on skin color and clinical factors, and calibration drift over short time intervals [10].

Hence, there is the need to improve BP measurement through PPG, and for this reason the interest is focused on the research of features of PPG signals that are correlated to BP and can be exploited for machine learning (ML) [15,16,17,18,19,20,21,22]. Features can be used for both non-invasive *SBP* and *DBP* measurements, and can be typically defined in time domain (including calculation of derivatives), in frequency domain or statistically. In addition, different ML methods have been investigated by researchers, such as support vector machines, regression trees, neural networks, linear regression, etc.

In this paper, the study and the research have been focused on the extraction of new and more significant features from PPG signals which have the potentiality of improving BP estimation. In particular, it has been found that by using the Maximal Overlap Discrete Wavelet Transform (MODWT) it is possible to enhance the PPG signal and to extract features that are important for this aim. As a matter of fact, it has been shown that MODWT improves the identification of characteristic points of the PPG signal, making it more similar to the ABP signal. Therefore, this work introduces several new features useful for BP estimation, to be extracted both from the enhanced and the base PPG signal, which have not been used in the literature. Another fundamental contribution of this work is the careful analysis of both new and known features with the aim of finding those most relevant for BP estimation. For this purpose, three different feature selection algorithms have been employed to analyze features extracted from a large number of PPG signals contained in the MIMIC-III Waveform Database. The analysis has led to the identification of the most informative features for BP estimation. This is an essential and general result that can be used in any other work involving the use of ML techniques for BP measurement from PPG signals. In fact, in an upcoming work our focus will be on ML models, to find the best algorithm using the significant features here individuated.

The paper is structured as follows: in Section 2 the source database and signal pre-processing are described; in Section 3 features extraction is presented, including new proposed features obtained by using MODWT; in Section 4 the error of characteristic points estimation from PPG signal is analyzed; the analysis of features is reported in Section 5, where most significant features are selected according to several criteria; and finally, in Section 6, there is a discussion of results, followed by the conclusion.

## 2. Dataset Pre-Processing and Labeling

**Figure 1:**
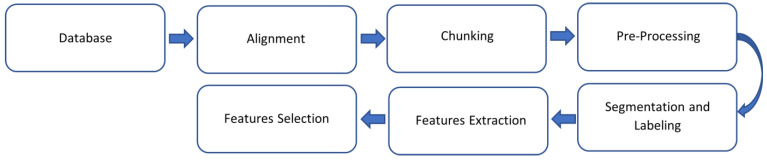
Workflow of data analysis.

The workflow of the data analysis is shown in Figure 1 and detailed in this and following sections.

In this work, a clinical dataset of physiologic signals acquired by the monitors of patients in an intensive care unit is used. However, since acquisitions are often interrupted or device configuration changed (due, e.g., to monitoring device failure, misconfiguration, priorities regarding patient condition, etc.), it is common that these datasets contain records of different lengths and signals may be missing or anomalous. Hence, many automatic checks have been made to discard unreliable data step-by-step as elaboration progresses. Data processing has been performed by using MATLAB R2022a.

### 2.1. Dataset

This study has been carried out using PPG and ABP signals available in the open access MIMIC-III Waveform Database [23,24,25], which has been selected because it is a very large, freely available database and an extension of the MIMIC II Waveform Database used in many other works about the analysis of biomedical signals. The MIMIC-III Waveform Database contains physiologic signals acquired from bedside monitors in adults aged 16 years or above (87% of admissions, 56% are male adults) and neonatal (13% of admissions) intensive care units (ICUs), and almost always includes one or more ECG signal and often ABP, PPG, and respiration signals. Age quartiles for adults are $Q_{1}=$ 52.8 years, $Q_{2}=$ 65.8 years and $Q_{3}=$ 77.8 years. The main patient diseases are coronary atherosclerosis of the native coronary artery (7.1%), unspecified septicemia (4.2%) and subendocardial infarction (initial episode of care) (3.6%). Data are collected with different medical devices of two critical care information systems: Philips CareVue Clinical Information System (with Intellivue MP-70 monitor) and iMDsoft MetaVision ICU. All protected health information in the database is deidentified and the demographic information is not provided. Moreover, PPG signals available in this database have been collected using a fingertip device, and all signals have been acquired at a sampling rate of 125 Hz.

Given the great size of the database, only part of folder #30 and the entire folder #32 were initially downloaded from the database, corresponding to 6740 patients; however, for the study presented in this paper, only patients where both PPG and ABP signals were available, corresponding to 1080 patients, have been considered. The WFDB MATLAB Toolbox [25,26] and purposely created functions have been used for data handling.

### 2.2. Alignment

**Figure 2:**
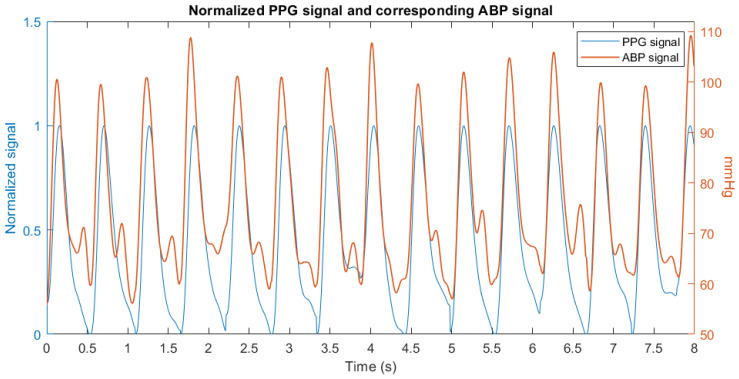
Alignment of the normalized PPG and ABP signals.

When ABP and PPG are acquired by different devices, they are provided without time alignment. Hence, in a preventive manner, such alignment has been carried out for all records using cross-correlation as reported in [16,27,28]. The cross-correlation function

(1) $$g\left(∆t\right)=\sum \mathrm{ABP}\left(t\right)\times \mathrm{PPG}\left(t+∆t\right)$$

has been computed between the two signals, then the location of the maximum value has been considered as time lead or lag. An example of the alignment is shown in Figure 2.

### 2.3. Chunking

For each record, consecutive blocks of 30 s have been obtained by signal chunking. That duration is arbitrary, but is considered adequate to obtain physiological information such as HR.

### 2.4. Pre-Processing

Then, PPG signals have been treated with: (1) denoising, by following the technique reported in [28,29,30] and used in [16], which consists of the application of a second-order Butterworth filter with pass-band 0.5 Hz to 8 Hz; (2) Z-score standardization, as carried out in [16]; and (3) baseline correction by means of the technique reported in [31,32,33] and used in [18], which consists of the removal of a fourth-order fitted polynomial. The obtained signal will be referenced later as $x_{FILT}$.

The ABP signal has also been processed to partition each chunk into pressure pulses, in which systolic blood pressure (*SBP*) and diastolic blood pressure (*DBP*) will be measured later. For that partitioning, a tenth-order lowpass Yule-Walker recursive filtering with an 8 Hz cut-off frequency has been applied to the ABP signal and the *slope sum function* has been calculated as reported in [34] to identify ABP pulses. The analyzing window length for the slope sum calculation is chosen as equal to the typical duration of the upslope of the pulse, and in this work it has been set to 0.128 s (similarly to [34]), which corresponds to 16 samples acquired at $f_{s}=125\;\mathrm{Hz}$.

Afterwards, the ABP pulses were evaluated according to the *complementary signal abnormality index* [34,35] and, if they exceed a threshold of 0.4, the entire ABP chunk and the corresponding PPG chunk were discarded from subsequent processing, as demonstrated in [27].

PPG chunks were also discarded if they failed a similarity test. In the literature, several similarity tests have been proposed [16,17], while in this paper a different test based on the coefficient of determination of several linear regressions has been used. For that purpose, PPG pulses have been individuated by using the corresponding bounds of ABP pulses, and then the following regressions have been calculated: of the PPG pulse on the corresponding ABP pulse; of a PPG pulse on the previous one; and of an ABP pulse on the previous one. In each one of the three cases, separately, the average of the coefficient of determination among the pulses of the chunk has been calculated and the chunk has been discarded if the average of the coefficient of determination was less than 0.8, as demonstrated in [27].

The coefficient of determination rsq has been computed as follows:

(2) $$\begin{array}{c} y_{resid}=x-y_{fit}\\ SS_{resid}=\sum_{i=1}^{N}y_{resid}{}^{2}\\ SS_{total}=\left(N-1\right)\;var\left(x\right)\\ rsq=1-\frac{SS_{resid}}{SS_{total}} \end{array}$$

where $x=\left[x_{1},\ldots ,x_{i},\ldots ,x_{N}\right]$ is the sampled PPG pulse signal of length N, with i=1,…,N, and $y_{fit}=\left[y_{1},\;\ldots \;,\;y_{i},\;\ldots \;,\;y_{N}\right]$ is the associated fitting.

### 2.5. Pulse Segmentation and Labeling

For the purpose of feature extraction, PPG pulses should be segmented independently of ABP pulses individuated in the previous subsection. To this end, in this work, the *slope sum function* calculation has also been extended to PPG in analogy to ABP, obtaining a partitioning of the chunk. The first obtained segment has been discarded because it may contain a partial pulse. For each PPG pulse, an additional signal quality index based on skewness has been calculated [19]. In this paper a PPG pulse has been discarded when skewness was less than zero.

Afterwards, PPG pulses were labeled with SBP and DBP values, necessary for the phase of features selection. For that purpose, ABP pulses have been re-segmented according to the boundaries of PPG pulses. Then, for each processed ABP pulse, *SBP* has been measured as the peak value and *DBP* as the next minimum value.

As a last check, ABP pulses and the corresponding PPG pulses were discarded if they had non-physiological values, namely when SBP>300 mmHg or DBP<20 mmHg.

## 3. Features Extraction

The extraction of the features has been carried out on PPG pulses or PPG chunks, processing the following signals:

1. $x_{FILT}$, obtained after the baseline correction;
2. $x_{NORM}$, obtained after normalization of $x_{FILT}$ in the range [0;1] for each pulse separately;
3. $x_{MODWT}$, obtained from $x_{FILT}$ after the MODWT enhancement that will be discussed later in this section.

The total number of extracted features is 195. The most relevant ones are listed and defined in Appendix S1 in the Supplementary Materials, distinguishing features already known in the literature [18,20,22] from features proposed in this paper for BP measurement on PPG signals.

Several proposed features have been extracted from individual pulses using the MATLAB built-in function *extract*, which gives features in the time domain and frequency domain on each pulse of $x_{FILT}$ and $x_{MODWT}$ and, in a few cases, also of $x_{NORM}$. Clearly, the definition of these features is well known; however, their application to $x_{MODWT}$ PPG signals to evaluate their significance for BP is novel.

Many features have been extracted from the PPG pulses on $x_{FILT}$ following [18,20,21,22], and are related to the amplitude of characteristic points, area, width, time, first and second derivative, nonlinear functions of features, and statistics.

**Figure 3:**
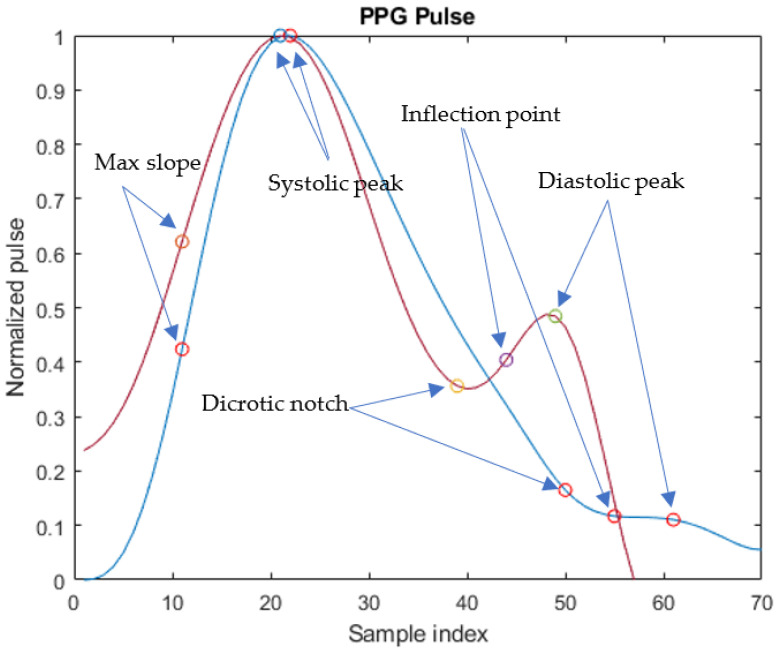
Different morphologies of PPG pulses of $x_{NORM}$ with characteristic points for two different patients: (blue pulse) adult patient; (red pulse) neonate patient. Sampling frequency is 125 Hz.

Moreover, the characteristic points have been identified on each PPG pulse of $x_{FILT}$, shown in Figure 3, and they are *max slope point* (MSP), *systolic peak* (SP), *dicrotic notch* (DN), *inflection point* (IP), and *diastolic peak* (DP).

The identification of those characteristic points cannot be obtained reliably based only on PPG amplitude since signal morphology changes from patient to patient and, in particular, it is related to the patient’s age and condition, as shown in Figure 3. Indeed, the DP may not be evident in the signal. Hence, points detection has been carried out based on the algorithms in [21] that also consider the first and second derivative of PPG pulses.

In this paper, an approach for the determination of characteristic points, alternative to [21], is proposed. It is based on the application of proper wavelet filtering to obtain an enhanced PPG signal that better resembles the ABP signal. For this purpose, the MODWT has been applied to each pulse, computed down to level $\log_{2}N$, where N is the number of samples of the pulse.

Wavelets have found application for the enhancement and denoising of medical images and bio signals [36,37,38]. In particular, MODWT has been used successfully for other physiological signals such as ECG, electroencephalogram (EEG), and magnetoencephalography (MEG) [39,40], but has also proved suitable, in our work, for the PPG signal. This wavelet is an undecimated wavelet transform similar to the discrete wavelet transform (DWT); however, no down sampling of coefficients is operated for its computation, hence it has a high amount of redundancy. Moreover, MODWT is a linear filtering operation and can be used to evaluate the behaviors of the signal dependent from the scale, and it is a time shift-invariant method and provides the alignment of the decomposed wavelet and scaling coefficients at each level with the initial signal; besides it allows the analysis of the localized signal variation. Therefore, there is a linear combination of scaling function and wavelet function considering the number of levels of the decomposition. The implementation of the MODWT, used in this work, performs the circular convolution in the Fourier domain.

**Figure 4:**
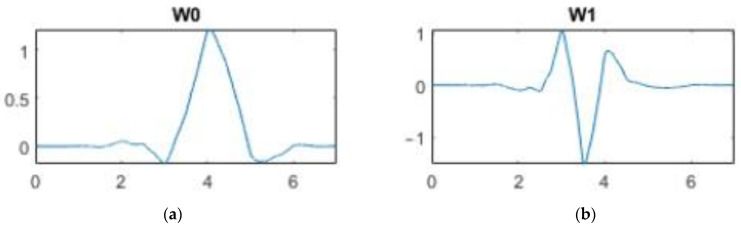
(**a**) Scaling function; (**b**) wavelet function.

The reconstruction of the pulse has been made using the Inverse Maximal Overlap Discrete Wavelet Transform (IMODWT). Among many MODWT variants, the *sym4* synthesis wavelet has been chosen because it provided better results in highlighting characteristic points. This wavelet’s family is near symmetric, orthogonal, and biorthogonal, with the scaling function and the wavelet function shown in Figure 4.

For the signal synthesis with IMODWT, only the fourth and the fifth levels coefficients have been used. As reported in [41], these selected coefficients maximize the energy of the QRS complex in ECG signals; in this work, the same approach has allowed the obtention of a frequency-localized version of the PPG pulse, which covers the bandwidth that maximizes its energy.

In fact, in this paper a key point is the use of MODWT on the PPG pulse that has allowed emphasis of the DP and the DN, which are two of the most relevant points of a PPG pulse, as seen in Figure 3. In the algorithm implemented in this research, SP is identified as the first peak of the enhanced PPG pulse, followed by a local minimum corresponding to DN, and then by a local maximum that is identified as DP.

**Figure 5:**
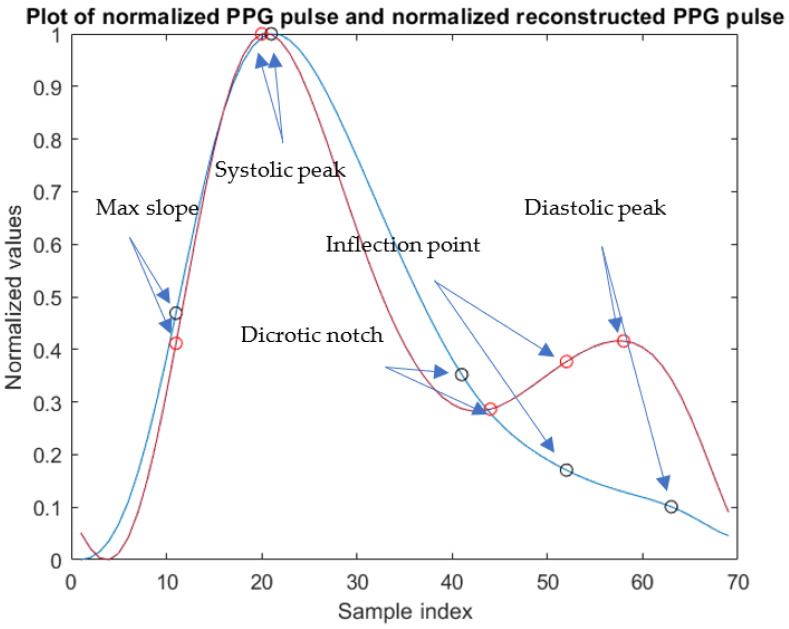
Pulse PPG before (blue) and after (red) the use of MODWT.

**Figure 6:**
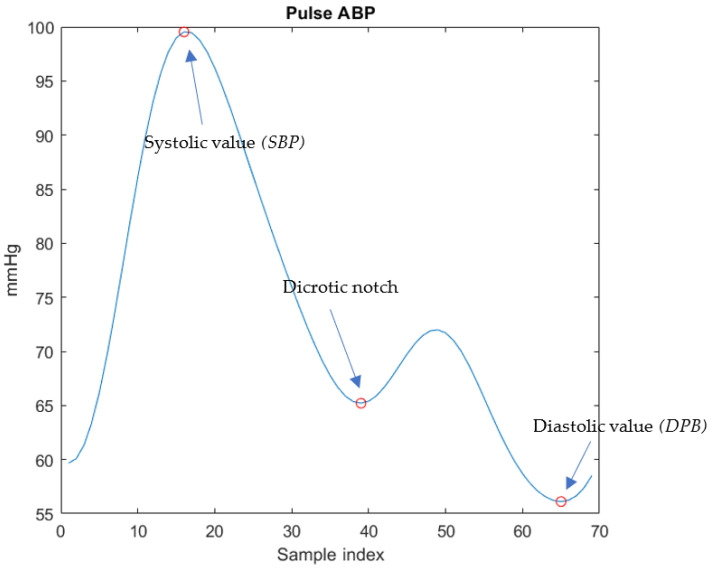
ABP pulse corresponding to the PPG pulse with SBP and DBP values.

As is evident in Figure 5, the DP and the DN are more pronounced in the enhanced signal and a greater similarity with the morphology of the ABP pulse shown in Figure 6 is evident. In the PPG pulse before the use of MODWT, instead, such points are less marked and cannot be reliably identified unless information from the first and second derivatives is also used.

From these new pulses, which show an improved similarity with the ABP pulse, additional features have then been extracted, including the characteristic points of the PPG pulse, time-related features, area-related features, energy related features, amplitude related features, and other features in time and frequency domain using the built-in function *extract* of MATLAB. Several features, extracted from the enhanced pulses, are reported in Appendix S1 in the Supplementary Materials.

Other features have been computed, considering the signal in time intervals that include couples constituted by a pulse and the next one, rather than a single pulse. In that case, however, the value calculated for each couple is averaged among all the couples of a chunk, and the resulting feature value is then assigned to each pulse of the chunk (e.g., Mean of tb1, Mean of tb2, Mean of TP/p2pi, Mean of TDN/p2pi, etc.).

Finally, there are features that have been calculated on the PPG chunk as a whole, rather than smaller intervals or single pulses. Their value is assigned, again, to each pulse of the chunk (e.g., Area from 2 to 5, Peak1, Freq1, Freq2, etc.). Several frequency related features have been obtained by means of the Fast Fourier Transform (FFT) applied to each PPG chunk of $x_{FILT}$ in order to extract the sixteen FFT features described in [18]. Other statistics-related features, such as mean and standard deviation, have been calculated on PPG chunks after denoising and before Z-scores standardization.

From the obtained set of $20\times 10^{6}$ pulses, each one described by its features, random samples have been extracted for the analysis performed in the next sections.

## 4. Error Analysis of SP, DP and DN Characteristic Points Estimation

Since the timing of the characteristic points of PPG pulses is particularly relevant, features based on them are correlated with pressure pulse transit times, stiffness of the large arteries, BP, and age [42].

In fact, the SP time (TSP) is due to pressure wave arrival from the left ventricle, while DP time (TDP) is due to a pressure wave that reaches lower limbs and is reflected back towards fingers, hence the time interval between them is related to transit times and also to arterial stiffness SI

(3) $$SI=\frac{h}{\left(TDP-TSP\right)}$$

where h is the patient’s height.

For that reason, it has been deemed necessary to study the difference of instants of SP (and also DP and DN) obtained by applying the algorithms in [21], based on derivatives, minus the ones obtained with the algorithm reported in this paper that exploits the MODWT, for which timings have been calculated, in both cases, with resolution 8 ms, which is equal to the sampling step (given the 125 Hz sampling frequency of signals in the dataset).

Therefore, a statistical analysis has been carried out on those errors by taking a random sample of $2\times 10^{6}$ pulses.

A first result is that the DP is visible in less than 1% of $x_{FILT}$ pulses, but in 95% of $x_{MODWT}$ pulses. Hence, in the vast majority of $x_{MODWT}$ pulses, the DP can be found directly by searching the second local peak (after the SP); instead, in the corresponding $x_{FILT}$, when the DP is not visible as a peak, the DP location commonly assumed in the literature is the one where the second derivative is zero, as also followed in this paper.

**Figure 7:**
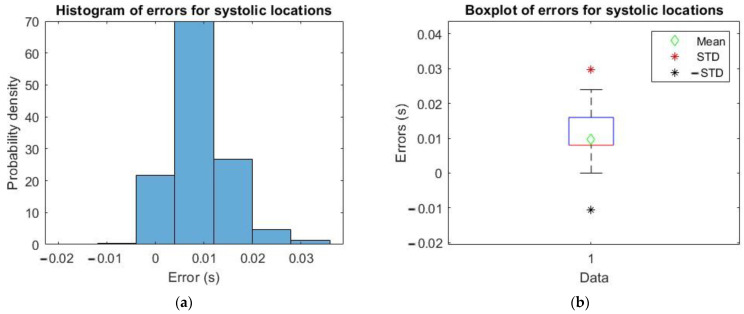
(**a**) Histogram of errors for systolic locations; (**b**) boxplot of errors with mean, mean + std and mean − std.

**Figure 8:**
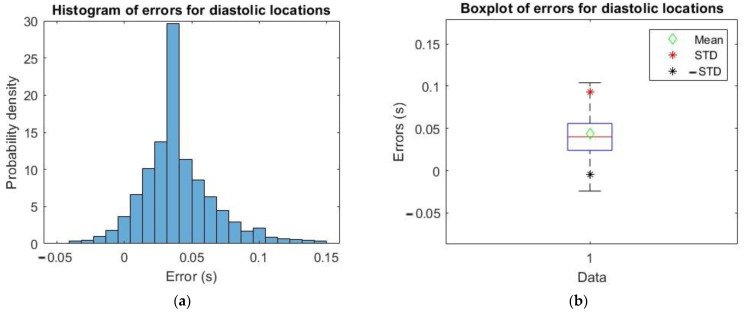
(**a**) Histogram of errors for diastolic locations; (**b**) boxplot of errors with mean, mean + std and mean − std.

**Figure 9:**
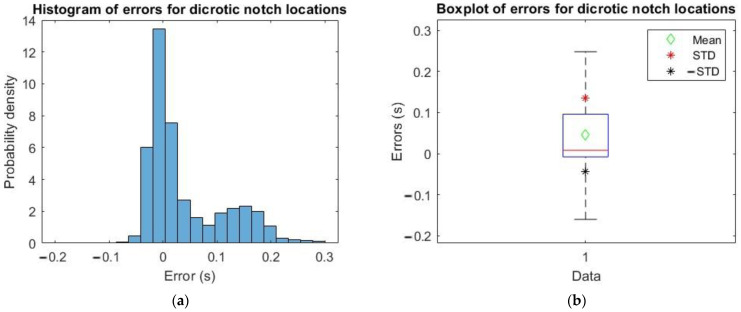
(**a**) Histogram of errors for dicrotic notch locations; (**b**) boxplot of errors with mean, mean + std and mean − std.

The location differences for $x_{FILT}$ minus $x_{MODWT}$ are shown in Figure 7, Figure 8 and Figure 9.

In Table 1, the mean and std of errors computed as described above are reported for the three characteristic points.

Therefore, from what is possible to observe from the plots in Figure 7, Figure 8 and Figure 9, and values in Table 1, the time error calculated in all three cases is very small, so the implemented algorithm based on MODWT is a valid alternative compatible with the one in the literature to identify the three characteristic SP, DP and DN points of the PPG pulse.

In the next section, we will analyze whether the features extracted using these three characteristic points obtained after MODWT enhancement, as well as the rest of the newly obtained features, are significant for BP measurement.

## 5. Features Selection

Subsequently to feature extraction, a selection of the most significant features related to *DBP* and *SBP* labels was made. A subset of 50,000 pulses has been randomly extracted for that analysis.

For these purposes, three different methods have been used following [18]. The algorithms used are: the Correlation-based Feature Selection (CFS), that calculates the correlation and selects features that are at the same time highly correlated with the label and negligibly correlated with each of the other features; the RReliefF [43,44], that penalizes the predictors that give different values to neighbors with the same labels, and rewards predictors that give different values to neighbors with different labels; and the MRMR (Minimum Redundancy Maximum Relevance) [45,46], that finds an optimal set of features that are mutually as dissimilar as possible and can effectively represent the label. This last algorithm minimizes redundancy among a feature set and maximizes the relevance of a feature set to the label.

**Figure 10:**
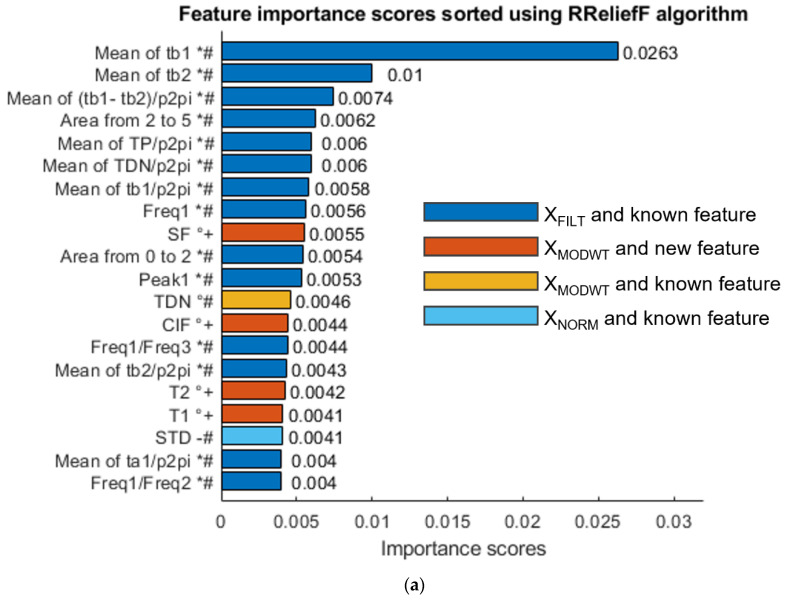

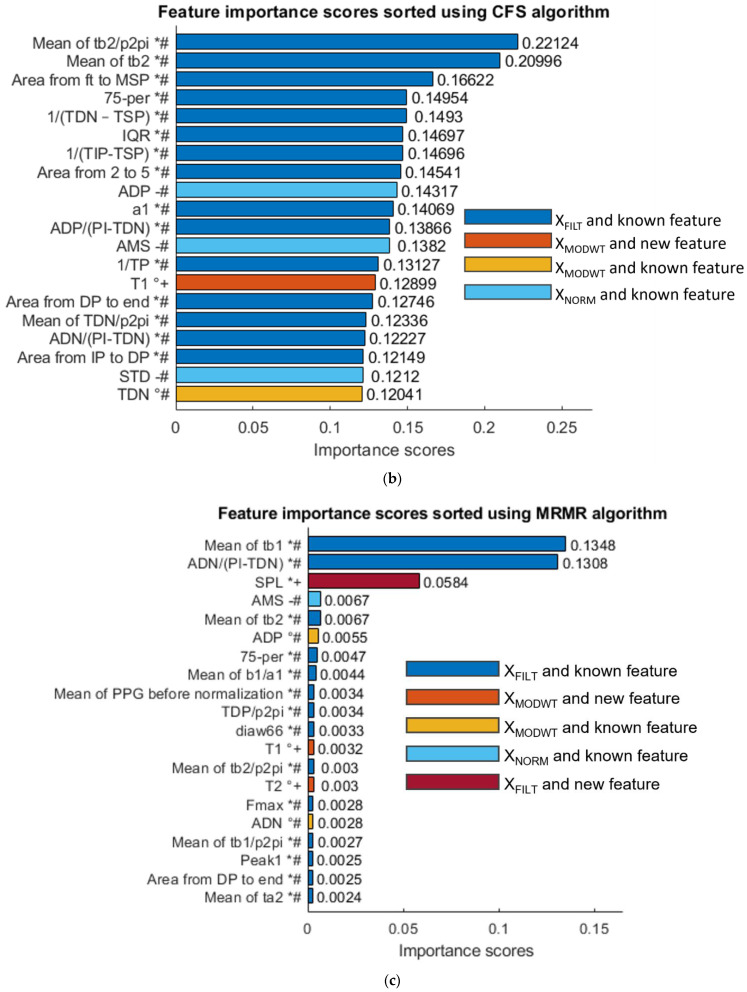
(**a**) Feature importance scores sorted using RReliefF algorithm for SBP measurement; (**b**) feature importance scores sorted using CFS algorithm for SBP measurement; (**c**) feature importance scores sorted using MRMR algorithm for SBP measurement. Feature labels are noted as follows: (*) calculated on $x_{FILT}$ (i.e., before MODWT enhancement), (°) calculated on $x_{MODWT}$ (i.e., after MODWT enhancement), (-) calculated on the normalized signal $x_{NORM}$, (+) new feature and (#) already known feature.

Using the three methods, the best features for *SBP* measurement have been selected. The first 20 features (out of 195) are shown in Figure 10 and listed in Table S1 in the Supplementary Materials.

The results show that the three methods select, among the best features, some of the new features extracted from the MODWT enhanced signal, which confirms their significance, as will be discussed in the next section.

**Figure 11:**
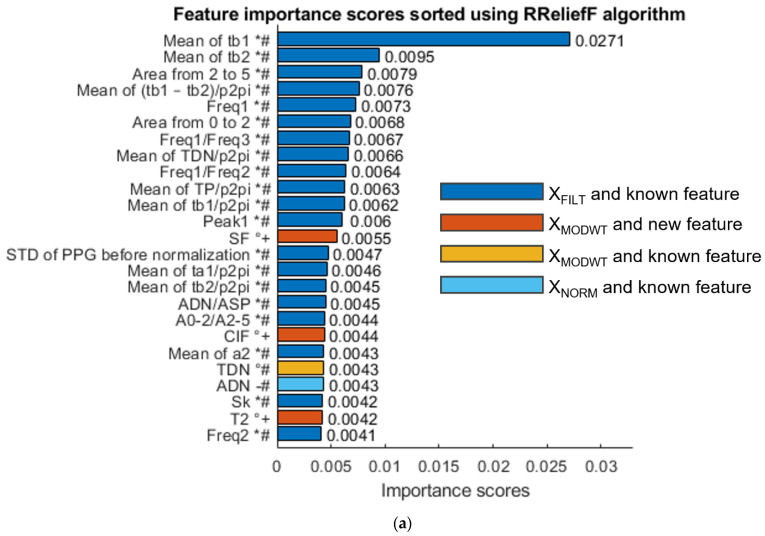

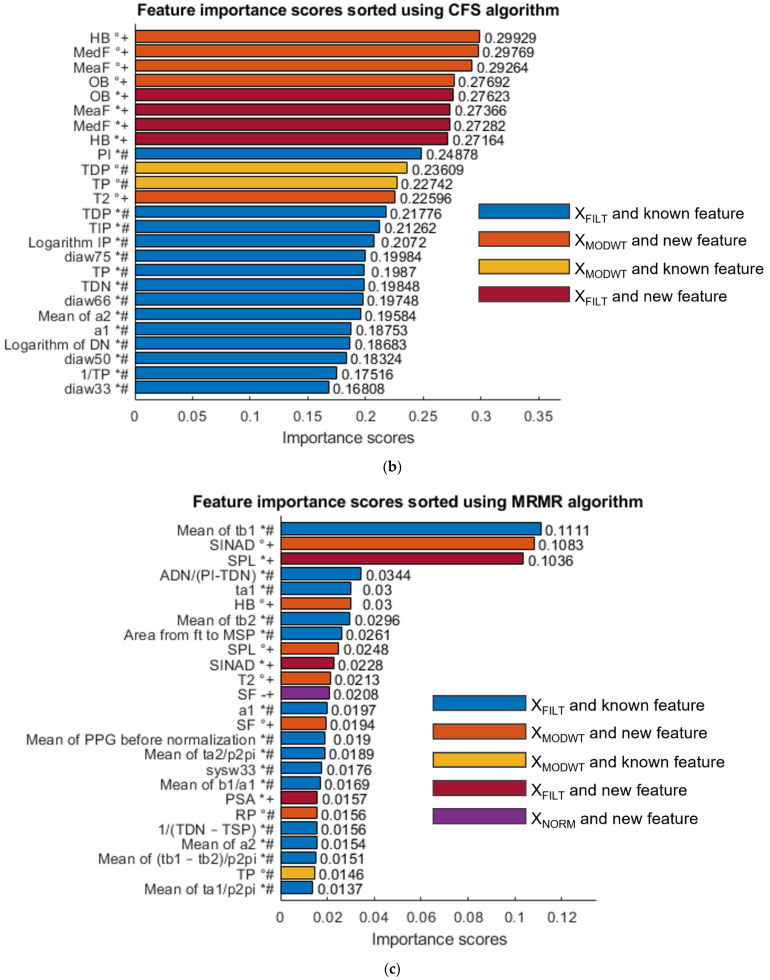
(**a**) Feature importance scores sorted using RReliefF algorithm for DBP measurement; (**b**) feature importance scores sorted using CFS algorithm for DBP measurement; (**c**) feature importance scores sorted using MRMR algorithm for DBP measurement. Feature labels are noted similarly to Figure 10.

The same procedure has also been followed to identify the most significant features associated with *DBP* measurement, as shown in Figure 11.

Therefore, as well, for the case in which the diastolic values are taken, the three methods select several newly defined features for both the MODWT enhanced PPG signal and the filtered signal ($x_{FILT}$).

## 6. Discussion

This section summarizes the results obtained during the features selection phase to underline that the features selected using the three methods, in both systolic and diastolic cases, include known features calculated after MODWT enhancement and new ones. Therefore, the following observations can be made.

In fact, several features have been extracted from the PPG signal and have been investigated using three selection methods; in particular, many already-known features have been calculated both before and after MODWT enhancement, showing that the use of MODWT alone leads to improvements. This happens for the following features: TDN, which is selected by CFS for *SBP* and by RReliefF for both *SBP* (0.0046 score) an *DBP* (0.0043 score); ADP and ADN selected by MRMR for *SBP*; TDP selected by CFS for *DBP*; and TP selected by both CFS and MRMR for *DBP*.

On the other hand, several new features have been selected even if they are not extracted from MODWT enhanced signals: SPL is selected by MRMR for *SBP*; OB, MeaF, MedF and HB are selected by CFS for *DBP*; and SPL, SINAD, SF and PSA are selected by MRMR for *DBP*.

Moreover, all selection criteria show that several proposed new features are more useful than already known features at exploiting MODWT enhancement. For example, SF is better than TDN according to RReliefF for *SBP*; T1 (score 0.12899 in Figure 10b) is better than TDN (score 0.12041) according to CFS for *SBP*; T1 (score 0.032 in Figure 10c) and T2 (score 0.0030) are better than ADN (score 0.028) according to MRMR for *SBP*; SF and CIF are better than TDN according to RReliefF for *DBP*; and many other features are better than TP and TDP according to CFS and MRMR for *DBP*.

Furthermore, features which are simultaneously calculated after MODWT enhancement and are new have frequently been selected. For example, SF and ClF are among the best features according to RReliefF for both *SBP* (Figure 10a) and *DBP* (Figure 11a). SF has also been selected by MRMR for *DBP*. Other features that have been selected by more than one method are T1 and T2.

In addition, it should be noted that many proposed features outperform already known features when their score is evaluated by CFS and MRMR for *DBP*, as shown in Figure 11b,c.

Considering the previously discussed results, the initial number of features, which was equal to 195, can be reduced by selecting the best significant features, shown in Table S1 in the Supplementary Materials. Several proposed features appear at least twice according to different selection methods, such as SF, ClF, T1, T2, SPL, TDN, TP, Area from DP to end, and so on. It should be noted that the shape factor SF, that has been proposed as a feature for PPG signal and is defined as the ratio between the RMS of the pulse and its mean absolute value, has been selected three times when applied to the enhanced signal $x_{MODWT}$. Moreover, the SF on the $x_{MODWT}$ is greater than SF on the $x_{FILT}$, since the DP is more pronounced in $x_{MODWT}$. That feature is related to the shape of the pulse, which depends strongly on vascular aging; hence it may also be relevant for arterial stiffness evaluation, which generally increases with age.

These considerations show that the MODWT enhancement of the PPG signal, as well as the individuated new features, lead to the extraction of significant information for BP that has the potential to improve its estimation through ML techniques.

## 7. Conclusions

In this paper, features of the PPG signal significant for BP measurements as well as the use of the MODWT to enhance the PPG signal have been discussed.

Even though the ABP signal allows a more direct and accurate measurement of BP, it is obtained in an invasive way, contrarily to PPG acquisition, which is easily and comfortably carried out, even at home. Hence, the use of PPG pulse enhancement by means of MODWT has been proposed, motivated by the fact that it shows greater similarity with the ABP pulse.

A first result presented in this paper is that the use of the enhanced signal allows a more reliable extraction of characteristic points of the PPG pulse, such as the DP and the DN. In fact, identifying the DP or the DN on PPG pulses is sometimes difficult, as they are less emphasized or missing according to patient’s age and condition. However, thanks to the proposed MODWT enhancement, characteristic points can be more directly obtained considering signal valleys and peaks, obtaining a performance comparable with the purposely defined algorithm presented in [21], as shown by error analysis performed in Section 4. For example, the DP was clearly marked in 95% of pulses (while it was only 1% for the untreated pulses), and that characteristic point had a 0.04 s mean error with respect to [21].

Indeed, as the second important result after features extraction and selection, several new proposed features (as well as old features) obtained from $x_{MODWT}$ and discussed in Section 6 have been found to be significant according to the scores given by three features evaluation methods, namely CFS, RReliefF and MRMR. It should be recalled that these methods permit the selection of optimal features that are relevant and not redundant for the estimation of the variables of interest, which in this case are the systolic and diastolic BP values. Therefore, the results reported in the paper can be used as a base to develop BP estimation methods based on PPG signal analysis.

## Figures and Tables

**Table 1:** Mean and std of errors for characteristic points.

	Mean (s)	Std (s)
Systolic Points (SP)	0.0097	0.0202
Diastolic Points (DP)	0.0441	0.0486
Dicrotic Notch Points (DN)	0.0458	0.0896

## Data Availability

Publicly available datasets were analyzed in this study. This data can be found in [24].

## Supplementary Materials

The following supporting information can be downloaded at: https://www.mdpi.com/article/10.3390/s23042321/s1, Appendix S1 listing more relevant signal features.
