# Supplementary material for: Photoplethysmography Signal Wavelet Enhancement and Novel Features Selection for Non-Invasive Cuff-Less Blood Pressure Monitoring
Source: Sensors (Basel). 2023 Feb 19;23(4):2321. doi: 10.3390/s23042321 (PMC9960464; doi:10.3390/s23042321)
Supplement: Supplementary file 1 [file sensors-23-02321-s001.zip › sensors-2074911-SI.pdf]

sensors

Suppplementary material

Photoplethysmography signal wavelet enhancement and novel features selection for non-invasive cuff-less blood pressure monitoring

Filippo Attivissimo, Luisa De Palma, Attilio Di Nisio*, Marco Scarpetta and Anna Maria Lucia Lanzolla

Appendix S1

Table S1 below defines the more relevant features calculated on PPG signals, which are in the set of selected features shown in Section 5. They are grouped by domain (e.g. energy, width-related, amplitude related, …) and they are noted as: (*) calculated on x_{FILT} (i.e. before MODWT enhancement), (°) calculated on x_{MODWT} (i.e. after MODWT enhancement), (-) calculated on the normalized signal x_{NORM}, (+) new feature and (#) already known feature.

In the table the following notation has been used: f_s is signal sampling frequency; $\boldsymbol{x} = [x_1, \dots, x_i, \dots, x_N]$ is the sampled PPG pulse signal of length N, with $i = 1, \dots, N$; $MODWT\{\boldsymbol{x}\}_{lk}$ is the MODWT wavelet coefficient up to scale 2^L, with $L = \lfloor \log_2 N \rfloor$, calculated at scale 2^l, with $l = 1, \dots, L$ and location k, with $k = 1, \dots, N$; X is the PPG chunk.

Table S1. Features.

Symbol	Definition	Notes	
**Energy-related features**			
$sigN2$	Squared signal Energy $sigN2 = \sum_{i=1}^{N} \lvert x_i \rvert^2$	*	+
			–
$wtN2$	Squared signal energy obtained from summing the wavelet coefficients over all levels $wtN2 = \sum_{l=1}^{L} \sum_{k=1}^{N} \lvert MODWT\{\boldsymbol{x}\}_{lk} \rvert^2$	°	+
**Width-related features**			
w	Width of the pulse $w = \frac{N}{fs}$	* °	#
$diaw33$	Width from DP to the subsequent intersection of $\boldsymbol{x}$ with level $0.33 \times ASP$	*	#
$diaw50$	Width from DP to the subsequent intersection of $\boldsymbol{x}$ with level $0.50 \times ASP$	*	#
$diaw66$	Width from DP to the subsequent intersection of $\boldsymbol{x}$ with level $0.66 \times ASP$	*	#
$diaw75$	Width from DP to the subsequent intersection of $\boldsymbol{x}$ with level $0.75 \times ASP$	*	#
$sysw33$	Width from intersection of $\boldsymbol{x}$ with level $0.33 \times ASP$ to SP	*	#

**Amplitude-related features**			
ASP	Amplitude of the systolic peak of $\boldsymbol{x}$	* °	#
ADP	Amplitude of the diastolic peak of $\boldsymbol{x}$	* ° -	#
ADN	Amplitude of the dicrotic notch of $\boldsymbol{x}$	* ° -	#
RP	$RP = \frac{ADP}{ASP}$ also known as the augmentation index	°	#
$D1$	$D1 = ASP - ADN$	°	+
$D2$	$D2 = ADP - ADN$	°	+
RD	$RP = \frac{D1}{D2}$	°	+
AMS	Amplitude of the max slope point (MSP) of $\boldsymbol{x}$	* -	#
$\frac{ADN}{ASP}$	Ratio between ADN and ASP of $\boldsymbol{x}$	*	#
**Time-related features**			
TP	Time interval from DP to the SP of $\boldsymbol{x}$	* °	#
TSP	Time interval from x_1 to the SP of $\boldsymbol{x}$	* °	#
TDP	Time interval from x_1 to the DP of $\boldsymbol{x}$	* °	#
TDN	Time interval from x_1 to the DN of $\boldsymbol{x}$	* °	#
TIP	Time interval from x_1 to the IP of $\boldsymbol{x}$	*	#
$T1$	Time interval from the DN to the SP of $\boldsymbol{x}$	°	+
$T2$	Time interval from the DP to the DN of $\boldsymbol{x}$	°	+
$p2pi$	The peak-to-peak interval is the distance between SP_{i+1} and SP_i	*	#
$Mean\ of\ \frac{TP}{p2pi}$	Mean of ratio between TP and $p2pi$ of $\boldsymbol{x}$	*	#
$Mean\ of\ \frac{TDN}{p2pi}$	Mean of ratio between TDN and $p2pi$ of $\boldsymbol{x}$	*	#
$\frac{1}{TDN - TSP}$	Reciprocal of difference between TDN and TSP of $\boldsymbol{x}$	*	#
$\frac{1}{TIP - TSP}$	Reciprocal of difference between time interval of IP (TIP) and TSP of $\boldsymbol{x}$	*	#
$\frac{ADP}{PI - TDN}$	The ratio of ADP to the differences between pulse interval (PI) and TDN of $\boldsymbol{x}$	*	#
$\frac{1}{TP}$	Stiffness of the large arteries: the inverse of TP of PPG pulse	*	#
$\frac{ADN}{PI - TDN}$	The ratio of AND to the differences between PI and TDN of $\boldsymbol{x}$	*	#
$\frac{TDP}{p2pi}$	Ratio of TDP and $p2pi$ of $\boldsymbol{x}$	*	#
PI	Pulse interval of $\boldsymbol{x}$ is the distance between x_1 and x_N	*	#
**Area-related features**			
$A1$	Area under the curve from $x_1$to the DN of $\boldsymbol{x}$	* °	#
$A2$	Area under the curve from DN to x_N	* °	#
RA	$RA = \frac{A2}{A1}$	°	+
$Area\ from\ x_1\ to\ MSP$	Area under the curve from x_1 to MSP of $\boldsymbol{x}$	*	#
$Area\ from\ IP\ to\ DP$	Area under the curve from IP to DP of $\boldsymbol{x}$	*	#
$Area\ from\ DP\ to\ x_N$	Area under the curve from DP to x_N of $\boldsymbol{x}$	*	#
**Statistic features**			
$Mean$	Mean of $\boldsymbol{x}$ $\mu = \frac{\sum_{i=1}^{N} x_i}{N}$	* ° -	#
STD	Standard deviation of $\boldsymbol{x}$	* ° -	#

	$\sigma = \sqrt{\frac{\sum_{i=1}^{N}(x_i - \mu)^2}{N-1}}$	
$75 - per$	75-th percentile of $\boldsymbol{x}$	* #
IQR	Inter Quartile Range of $\boldsymbol{x}$ $IQR = Q_3 - Q_1$ where Q_3 is the third quartile and Q_1 is the first quartile	* #
Mean of PPG before standardization	Mean of PPG signal before standardization	* #
STD of PPG before standardization	STD of PPG signal before standardization	* #
Sk	Skewness of $\boldsymbol{x}$ $Sk = \frac{\frac{\sum_{i=1}^{N}(x_i - \mu)^3}{N}}{\sigma^3}$	* #
**Time domain features**		
RMS	Root mean square $RMS = \sqrt{\frac{\sum_{i=1}^{N}\lvert x_i\rvert^2}{N}}$	* ° - +
SF	Shape factor of pulse $SF = \frac{RMS}{MAV}$ where MAV is the mean absolute value $MAV = \frac{\sum_{i=1}^{N}\lvert x_i\rvert}{N}$	* ° - +
SNR	Signal to noise ratio $SNR = \frac{P_x}{P_n}$ where P_x is the power of $\boldsymbol{x}$ and P_n is the power of noise	* ° +
THD	Total harmonic distortion $THD = \sqrt{\sum_{j=2}^{n_h}\frac{h_j}{h_1}} \times 100$ where h_1 is the power at the fundamental frequency, h_j is the power at the j-th harmonic component, and n_h is the number of components	* ° +
$SINAD$	Signal to noise and distortion ratio in decibels $SINAD = 20\log\frac{P_x}{(P_n + P_d)}$ where P_d is the power of distortion	* ° +
CrF	Crest factor: $CrF = \frac{ASP}{RMS}$	* ° +
ClF	Clearance factor: $ClF = \frac{ASP}{\left(\frac{\sum_{i=1}^{N}\sqrt{\lvert x_i\rvert}}{N}\right)^2}$	* ° - +
IF	Impulse factor: that is the ratio between ASP and MAA $IF = \frac{ASP}{MAA}$ where MAA is the mean of the absolute amplitude	* ° - +
**Frequency domain features**		

$MeaF$	Mean frequency of the power spectrum of $\boldsymbol{x}$	* ○	+
$MedF$	Median frequency of the power spectrum of $\boldsymbol{x}$	* ○	+
AP	Average band power of $\boldsymbol{x}$	* ○	+
OB	Occupied bandwidth at 99% of $\boldsymbol{x}$	* ○	+
HB	Half-power bandwidth at 3 dB of $\boldsymbol{x}$	* ○	+
PSA	Peak spectral amplitude of $\boldsymbol{x}$	* ○	+
SPL	Spectral peak location of $\boldsymbol{x}$	* ○	+
**Features related to the first and second derivative**			
$a1$	The first maximum peak from the first derivative of $\boldsymbol{x}$	*	#
$a2$	The first maximum peak from the second derivative of $\boldsymbol{x}$ after $a1$	*	#
$b1$	The first minimum peak from the first derivative of $\boldsymbol{x}$ after the $a1$ occurred	*	#
$b2$	The first minimum peak from the second derivative of $\boldsymbol{x}$ after $a2$	*	#
$ta1$	The time interval from x_1 to the time at which $a1$ occurred of $\boldsymbol{x}$	*	#
$ta2$	The time interval from x_1 to the time at which $a2$ occurred of $\boldsymbol{x}$	*	#
$tb1$	The time interval from x_1 to the time at which $b1$ occurred	*	#
$tb2$	The time interval from x_1 to the time at which $b2$ occurred	*	#
$Mean\ of\ tb1$	Mean of the time intervals from x_1 to the time at which $b1$ occurred	*	#
$Mean\ of\ tb2$	Mean of the time intervals from x_1 to the time at which $b2$ occurred	*	#
$Mean\ of\ \frac{tb1 - tb2}{p2pi}$	Mean of the ratios of difference between $tb1$ and $tb2$ and $p2pi$	*	#
$Mean\ of\ \frac{tb1}{p2pi}$	Mean of ratio between $tb1$ and $p2pi$ of $\boldsymbol{x}$	*	#
$Mean\ of\ \frac{tb2}{p2pi}$	Mean of ratio between $tb2$ and $p2pi$ of $\boldsymbol{x}$	*	#
$Mean\ of\ \frac{ta1}{p2pi}$	Mean of ratio between $ta1$ and $p2pi$ of $\boldsymbol{x}$	*	#
$Mean\ of\ \frac{b1}{a1}$	Mean of the ratio between $b1$ and $a1$ of $\boldsymbol{x}$	*	#
$Mean\ of\ ta2$	Mean of $ta2$ of $\boldsymbol{x}$	*	#
$Mean\ of\ a2$	Mean of $a2$ of $\boldsymbol{x}$	*	#
$Mean\ of\ \frac{ta2}{p2pi}$	Mean of ratio between $ta2$ and $p2pi$ of $\boldsymbol{x}$	*	#
**FFT features**			
Area from 2 to 5	Area under the curve from 2 Hz to 5 Hz for the FFT of X	*	#
$Peak1$	The amplitude of the first peak from the FFT of X	*	#
$Freq1$	The frequency at which the first peak from the FFT of X occurred	*	#
$Freq2$	The frequency at second peak from the FFT of X	*	#
Area from 0 to 2	Area under the curve from 0 Hz to 2 Hz for the FFT of X	*	#
$\frac{Freq1}{Freq3}$	The ratio of $Freq1$ to the frequency at third peak from the FFT ($Freq3$) of X	*	#
$\frac{Freq1}{Freq2}$	The ratio of $Freq1$ to the frequency at second peak from the FFT ($Freq2$) of X	*	#

$Fmax$	The value of highest frequency in the spectrum of X	*	#
$\frac{Area\ from\ 0\ to\ 2}{Area\ from\ 2\ to\ 5}$	Ratio between $Area\ from\ 0\ to\ 2$ and $Area\ from\ 2\ to\ 5$	*	#
**Non-linear functions of features**			
Logarithm IP	Logarithm of location of IP (LIP) of $\boldsymbol{x}$<br>$Logarithm\ IP = \ln LIP$	*	#
Logarithm of DN	Logarithm of location of DN (LDN) of $\boldsymbol{x}$<br>$Logarithm\ DN = \ln LDN$	*	#
